# Simultaneous Application of Electron Beam Irradiation and Freezing as an Effective Method for Shelf Life Extension of Minced Turkey Meat

**DOI:** 10.1002/fsn3.4752

**Published:** 2025-01-12

**Authors:** Mahdieh Salari, Moein Khatami

**Affiliations:** ^1^ Department of Food Science and Technology, Faculty of Agriculture University of Tabriz Tabriz Iran; ^2^ Department of Food Science and Technology, Faculty of Technical and Engineering University of Science and Arts of Yazd Yazd Iran

**Keywords:** chemical and microbial quality, electron beam irradiation, freezing storage, minced Turkey meat, sensory properties

## Abstract

In this study, minced turkey meat samples were subjected to electron‐beam irradiation with dosages of 0, 1.5, 3, and 5 kGy, and then microbial (mesophilic and psychrotrophic bacteria), physiochemical (pH, water activity [a_w_], thiobarbituric acid reactive substances [TBARS], and peroxide value [PV]), and sensory (color, odor, texture, and overall acceptability) analyses were performed at 0 and 6 months of freezing storage (−18°C). Results showed that by 5 kGy irradiation and freezing treatments, the counts of psychrotrophic and mesophilic bacteria reduced remarkably (*p* < 0.05) and reached from 4.44 to 2.05 and from 4.9 to 2.00 log cfu/g, respectively. pH and a_w_ of samples did not change notably after treatment and 6 months of freezing storage, the pH and a_w_ of the minced turkey meat were 5.74 and 0.95 for 5 kGy irradiated samples, respectively. Despite the increase of TBARS and PV values, electron‐beam irradiation at 1.5 and 3 kGy had no notable impact on the sensory characteristics of minced turkey meat. The obtained findings revealed that the simultaneous use of irradiation and freezing is a promising method for the increase of shelf life and preservation of minced turkey meat quality.

## Graphical abstract

Turkey meat was treated by electron‐beam irradiation and frozen storage (−18°C). Electron‐beam irradiation plus freezing (−18°C) treatments effectively decreased microbial contamination of turkey meat. The combined treatment did not have a negative effect on the chemical and sensory properties of turkey meat.

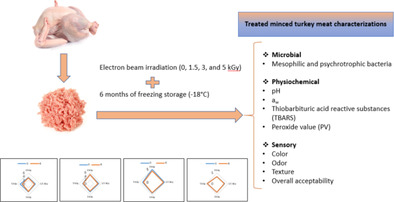

## 1 Introduction

Meat contains essential amino acids, minerals (iron and zinc), vitamins (B3 and B12), and high energy, and therefore it is one of the most valuable sources of protein and is among the best and most complete foods (Al‐Sheddy, Al‐Dagal, and Bazaraa 1999). The global consumption of meat is 280 million tons annually, and it is estimated that this amount will double by 2050 (Arokiyaraj, Dinakarkumar, and Shin 2024). Meat is classified into three main categories: red meat, poultry, and seafood. Red meat comes from livestock such as beef, pork, lamb, and goat. Poultry includes white meat from animals like chicken and turkey. Seafood includes fish, crustaceans (such as lobsters and crabs), and mollusks (such as oysters, clams, mussels, and scallops) (Khalid et al. 2023). Even though meat consumption in industrialized countries has stabilized at a considerable level, there have been significant changes in the type of meat consumption. In recent decades, global poultry meat consumption has significantly increased. Turkey meat, in particular, has emerged as the second most popular choice among poultry meats worldwide because of its unique sensory characteristics, including desirable texture, color, and taste, as well as its high nutritional value. It offers a high protein concentration, low fat content (lower than that of chicken, at approximately 1.21%), and is rich in essential micronutrients such as phosphorus, selenium, magnesium, zinc, potassium, and iron, which benefit the nervous system and immune health (Martínez‐Laorden, Arraiz‐Fernández, and González‐Fandos 2023; Solaesa et al. 2024). Additionally, turkey meat consumption aligns with diverse dietary preferences and is compatible with various religious customs. As a staple in Thanksgiving traditions, it can be readily incorporated into many diets. By 2020, global turkey meat production reached 6 million tons (Kálmán and Szőllősi 2023). However, turkey meat is highly perishable and spoils quickly against the activities of unfavorable microorganisms.

The goal of preserving food is to refrain from or postpone the occurrence of spoilage by external and internal factors, as a result of which the food remains usable for a certain time. Spoilage microorganisms and enzymes present in foods adversely affect their shelf life and safety. Among the physical methods of food preservation, irradiation is one of the most effective procedures for decontamination and destruction of food spoilage microorganisms, especially in meats and their products (Aymerich, Picouet, and Monfort 2008). Recently, irradiation of foodstuff, as a green process, has been studied in various research activities for enhancing the safety and durability of foods (Gupta, Guha, and Srivastav 2023). The nonthermal method of “irradiation technology” involves applying a particular product to levels of ionizing radiation that are controlled to destroy pathogenic microorganisms and improve food quality and finally contribute to a safer product for human consumption (Fallah, Sarmast, Ghasemi, et al. 2023; Jia et al. 2023). The use of this method is acceptable in 41 countries and over 100 food products. Annually, more than 500,000 tons of food products are irradiated, including dairy products, meats, eggs, sea products, fruits and vegetables, spices, and other dry foods (Kume et al. 2009). In this method, food products can be exposed to radiation during the final process while they are inside the package, and possible contamination is removed from them until they are taken out of the package and consumed. The procedure of electron‐beam irradiation is quick and does not result in radioactive waste (Black and Jaczynski 2006). Also, it kills most spoilage and pathogenic microorganisms. Therefore, it can be considered a preservation procedure for food products to decrease microbial enumeration (Rodriguez et al. 2006).

Freezing is a conventional method to protect and extend the storage time of food. In addition to preserving nutrients in food with the least change for a relatively long time, freezing can prevent the growth and development of pathogens and suppress their activity (ISO 2003). However, long‐term storage can cause cell and muscle fiber destruction because of the production of ice crystals in the tissue.

Low doses of ionizing radiation have been linked to a lack of microbial immunity and a negligible increase in product shelf life, according to previous studies (Fallah, Sarmast, Jafari, et al. 2023). However, using irradiation at higher approved doses leads to the formation of free radicals, which can oxidize into peroxides, degrade proteins, speed up lipid oxidation, cause vitamin loss, and result in undesirable flavors, colors, and odors (Brewer 2009). As a result, combining irradiation with other protective technologies not only enhances the microbiological safety and shelf life of the product but also mitigates the mentioned negative impacts.

Based on our investigations, no study has been done on the simultaneous use of electron‐beam irradiation and freezing methods on the quality, safety, and shelf life of minced turkey meat. Due to the potential for high doses of radiation to cause sensory changes in meat and pose risks to consumers, it is essential to research the optimal radiation dose for minced turkey meat to decrease microbial enumeration, prolong shelf life, and assess the commercial feasibility of using irradiation for minced turkey meat. Therefore, this research aims to examine the simultaneous effect of freezing storage and electron‐beam irradiation on the preservation of minced turkey meat to achieve the optimal dose and the highest possible storage time without causing significant changes to its chemical and sensory properties.

## 2 Materials and Methods

### 2.1 Materials

pH buffer 4 and 7, thiobarbituric acid (TBA), glacial acetic acid, trichloroacetic acid (TCA), chloroform, sodium thiosulfate, potassium iodide, plate count agar (PCA), and peptone water were purchased from Merck Co., (Germany). For all experiments, analytical‐grade chemicals were used.

### 2.2 Preparation of Turkey Meat

The turkey meat was purchased from a local butcher shop (Yazd, Iran), immediately after slaughter. After cleaning the visceral contents, the resulting meat was minced along with the skin. The samples were placed in polyethylene bags next to ice. The minced turkey meat was then divided into four groups (each weighing 100 ± 5 g). Three groups were irradiated, and the last group was considered as a control (nonirradiated).

### 2.3 Irradiation Process

The minced meat samples were placed in sterile polyethylene bags and then put inside the pallets related to the irradiation device MeV (TT2000, Belgium) and were affected by the accelerated electron‐beam at the Atomic Energy Agency of Yazd. The doses delivered were 1.5, 3, and 5 kGy, with a dose rate of 0.46 Gy/s, a maximum power of 100 kW, and an electron energy of 10 MeV. FWT dosimetry films were employed to measure the absorbed radiation. These films, which are used to assess the accuracy of the irradiation process, are initially colorless and thin, gradually turning blue as they absorb radiation. After irradiation, the samples were stored in an ice box and transported to the laboratory for further analysis.

### 2.4 Samples Storage

The irradiated and nonirradiated samples were kept in a freezer at −18°C ± 1°C and were subjected to microbial and chemical analysis at 0 and 6 months after storage time.

### 2.5 Microbial Characterization

Exactly 25 g of each sample was weighted under aseptic conditions and mixed to 225 mL of peptone water (0.1%) in Stomker and homogenized under an aseptic environment at 200 rpm for 1 min. Afterward, serial dilutions (10^−1^–10^−5^) were inoculated into the Petri dishes which contained PCA culture medium. Bacterial tests were included:

- Cultivation and counting of aerobic mesophilic bacteria by the standard plate culture method in PCA medium (incubation for 48 h at 37°C).
- Cultivation and counting of psychrotrophic bacteria by standard plate culture method in PCA medium (incubation for 10 days at 7°C).

The enumeration of bacteria was reported as colony‐forming units per gram of minced meat sample in a logarithm scale (log cfu/g) (Faradonbeh et al. 2024).

### 2.6 pH and Water Activity (a_w_)

The samples were homogenized (Heidolph Silent Crusher M, Germany) for 1 min at 9500 rpm in 20 mL of distilled water to test the pH. The mixture was kept for 10 min to settle. Before analysis, calibration of the pH meter (STARTER 3000, OHAUS Co., Switzerland) was performed by buffers 4 and 7 (Vidal et al. 2024). An a_w_ meter (ROTRONIC HC2‐AW‐USB, Switzerland) was used to determine the a_w_ parameter. Approximately, 5 g of samples were utilized for each test (25°C).

### 2.7 Peroxide Value Determination

The rate of fat oxidation of minced meat samples during storage was determined by peroxide value (PV) measurement. To determine the PV of the minced meat samples, the iodometric method described by Sahraee et al. (2020), was used. Briefly, 1 g of the sample (oil extracted from the meat) was dissolved in a chloroform and acetic acid mixture (3:2 v/v) under magnetic agitation. Then, 0.5 mL of saturated potassium iodide solution was added, and the mixture was left in the dark for 1 min. Next, 0.5 mL of 1% (w/v) starch reagent was added, and the solution was titrated with 0.01 N sodium thiosulfate. The PV (milliequivalents of peroxide per kg of sample) was then calculated using the following equation:

(1) $$\mathrm{PV}=\frac{1000\times S\times N}{W}$$

where *S* represents the volume of sodium thiosulfate consumed in the titration, *N* is the normality of the sodium thiosulfate solution, and *W* is the weight of the extracted oil, respectively.

### 2.8 Thiobarbituric Acid Reactive Substances

Using a modified version of the procedure outlined by Khatib et al. (2020), thiobarbituric acid reactive substances (TBARS) were evaluated to identify secondary lipid oxidation products. To summarize at first 20 g of sample turkey flesh and 20 mL of 10% w/v TCA solution were combined. Following 10 minutes of homogenization, the mixture was centrifuged at 10,000 g for 10 minutes at 4°C. After adding a 2.0 mL aliquot of the supernatant to a tube holding 2.0 mL of TBA solution (0.01 M), the tube was incubated for 20 min at 100°C in a water bath. The malondialdehyde (MDA) concentration (mg of MDA equivalent per kilogram of turkey flesh) was computed using the following equation after the absorbance was measured at 532 nm by a spectrophotometer (Pharmacia Biotech Co., England):

(2) $$\mathrm{mg}\;\mathrm{MDA}/1000\;g\;\text{sample}={\mathrm{Abs}}_{532}\times 5.4$$

### 2.9 Sensory Properties

Color, odor, texture, and overall acceptability of the treated and untreated samples were assessed after 0 and 6 months of freezing storage (−18°C). For this purpose, 20 trained panelists were hired and a 5‐point hedonic test (a score of 1 indicates very poor quality, whereas a score of 5 represents excellent quality) was applied. For odor and overall acceptability evaluation, the samples were cooked. 5 g of minced meat was placed into an Erlenmeyer flask containing distilled water and cooked on a direct flame until boiling, and then the odor of boiling was tested by panelists. The minced meat samples were named with three‐digit random numbers (Badr 2004).

### 2.10 Statistical Analysis

To analyze the data, a completely randomized design was used. The experiments were done in three replications at 0 and 6 months after storage. Utilizing SPSS 25 software (SPSS Inc., Chicago, IL, USA), means were compared using one‐way analysis of variance (ANOVA) and Duncan's multiple range test at a 5% error level. Graphs were created using Excel software.

## 3 Results and Discussion

### 3.1 Microbial Properties

#### 3.1.1 Mesophilic Bacteria

As detailed in Table 1, the preliminary enumeration of mesophilic bacteria in the nonirradiated minced meat samples was 4.44 log cfu/g, which was the highest among all treatments. As the radiation dose increased, the number of mesophilic bacteria decreased notably and reached 4.06, 2.74, and 2.70 cfu/g in 1.5, 3, and 5 kGy irradiated minced meat samples, respectively. This issue indicated the significant effect of radiation in destroying mesophilic bacteria and also the direct relationship between increasing the radiation dose and decreasing the enumeration of bacteria (*p* < 0.05). In addition, our findings showed that storage in freezing conditions causes a noticeable decrease in mesophilic bacteria enumeration (*p* < 0.05). In other words, in irradiated minced meat as well as the control samples, the highest and the lowest number of mesophilic bacteria was observed at 0 and after 6 months of freezing storage (−18°C), respectively. It is noteworthy that, due to the use of high radiation dosage, 3 and 5 kGy, the difference in the enumeration of mesophilic bacteria was greater between 0 and 6 months after storage, revealing the double impact of applying accelerated electron irradiation and freezing storage on the number of mesophilic bacteria. This indicates that the simultaneous use of irradiation and cryopreservation has a greater effect on reducing the population of mesophilic bacteria, which is consistent with the basic concept of Hurdle technology. This technology was developed to reduce the use of preservatives in foods and involves the use of a combination of traditional and innovative preservation techniques to create an additional antimicrobial effect, thereby improving food quality (Giannakourou et al. 2023).

**TABLE 1:** Numbers of mesophilic bacteria (log cfu/g) in control and electron‐beam irradiated minced turkey meat samples after storage at −18°C.

Irradiation doses (kGy)	Storage time (months)	
	0	6
0	4.44 ± 0.01^Aa^	4.29 ± 0.03^Ab^
1.5	4.06 ± 0.015^Ba^	4 ± 0.01^Bb^
3	2.74 ± 0.011^Ca^	2.43 ± 0.015^Cb^
5	2.70 ± 0.05^Da^	2.05 ± 0.01^Db^

*Note:* Different capital letters in each column indicate significant differences among treatments, and different small letters in each row indicate significant differences in storage time (*p* < 0.05).

The World Health Organization (WHO) states that radiation‐treating food at appropriate dosages (< 10 kGy) is a safe and effective way to increase food safety and shelf life without compromising the meal's nutritional value or sensory appeal (Organization 1999). Utilization irradiation for meat and meat‐based products is a technology that improves their shelf life by destroying microorganisms. The inactivation of microorganisms is thought to be initiated by ionizing radiation and is because of the inactivation of DNA (Arokiyaraj, Dinakarkumar, and Shin 2024). The properties of the desired food, radiation intensity, the condition and type of the radiation source, and the microorganisms' type can affect the efficiency of food irradiation (Fallah, Sarmast, Ghasemi, et al. 2023). Similar results were also obtained by Badr (2004) who demonstrated that rabbit meat irradiation at 3 kGy reduces the population of *Staphylococcus aureus* , *Listeria monocytogenes* , *Enterobacteriaceae*, and *Enterobacter faecalis* by more than 3, 2, 4, and 1.4 log cfu/g, respectively. It has been shown that no viable microorganisms were found in turkey meat samples after electron‐beam irradiation with a dose of 2 kGy (Bliznyuk et al. 2022). In another study, irradiation of gamma ray at doses of 0, 1.5, 3, and 4.5 kGy resulted in the inactivation of *E. coli* , *S. typhimurium* , and *L. monocytogenes* in chicken products. So that even after 15 days of storage, this product still had pleasant sensory and texture characteristics (Hayam 2013).

#### 3.1.2 Psychrotrophic Bacteria

The enumerations of psychrotrophic bacteria for minced turkey meat samples during the freezing storage are detailed in Table 2. In the control sample (nonirradiated), the number of psychrotrophic bacteria was 4.89 log cfu/g. However, by increasing the irradiation dose, the number of psychrotrophic bacteria decreased significantly and reached 2.2 log cfu/g for a 5 kGy irradiated sample, which indicates the significant impact of irradiation treatment on reducing the enumeration of psychrotrophic bacteria (*p* < 0.05). The results revealed that after 6 months of freezing storage, the count of psychrotrophic bacteria in all samples decreased notably (*p* < 0.05). In general, irradiation caused a remarkable reduction in the psychrotrophic bacteria population. Also, freezing storage has played a role as an auxiliary factor in reducing the population of these bacteria. The observed results were in agreement with the findings obtained in Section 3.1.1.

**TABLE 2:** Numbers of psychrotrophic bacteria (log cfu/g) in control and electron‐beam irradiated minced turkey meat samples after storage at −18°C.

Irradiation doses (kGy)	Storage time (months)	
	0	6
0	4.89 ± 0.01^Aa^	4.46 ± 0.015^Ab^
1.5	4.06 ± 0.02^Ba^	3.33 ± 0.001^Bb^
3	3.23 ± 0.02^Ca^	2.3 ± 0.011^Cb^
5	2.2 ± 0.01^Da^	2.00 ± 0.005^Db^

*Note:* Different capital letters in each column indicate significant differences among treatments, and different small letters in each row indicate significant differences in storage time (*p* < 0.05).

The study of Al‐Bachir and Zeinou (2009) showed that gamma ray irradiation at doses of 2, 4, and 6 kGy can reduce the total population of aerobic microorganisms in minced camel meat by up to 3, 4, and 5 log cycles, respectively. They also stated that the population of coliforms in samples decreased from 10^3^ cfu/g in the nonirradiated sample to < 10 cfu/g in gamma‐irradiated samples. Also, the findings of this research are consistent with the results of Gomes et al. (2003). These researchers reported that chicken fillets irradiation by gamma ray at 3 and 4 kGy caused a notable decrease in the count of psychrotrophic bacteria. Another study demonstrated that gamma irradiation of smoked guinea fowl meat at 2.5–7.5 kGy significantly increases the meat shelf life and quality by up to 7 weeks under refrigerator temperature (Otoo, Ocloo, and Appiah 2022).

### 3.2 pH

**FIGURE 1:**
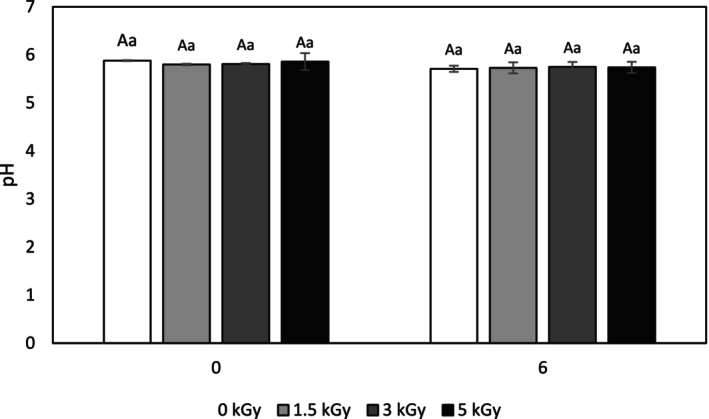
pH of control and electron‐beam irradiated minced turkey meat samples after 6 months storage at −18°C. Different capital letters indicate significant differences among treatments and different small letters indicate significant differences in storage time (*p* < 0.05).

The determined pH data are shown in Figure 1. It was observed that the pH of the untreated minced meat sample was 5.78 at time 0 and decreased with the irradiation process, although this reduction was not remarkable (*p* < 0.05). Therefore, the findings revealed that the irradiation process has no notable impact on the pH of minced turkey meat, which is in line with previous studies (Kanatt, Chawla, and Sharma 2015; Rodrigues et al. 2020; Sales et al. 2020). It is proven that irradiation does not have a notable influence on the pH value of poultry and meat (Hashim, Yusop, and Rahman 2024).

After 6 months of freezing storage, the pH of the minced meat decreased slightly and reached 5.71, 5.73, 5.75, and 5.74 for nonirradiated and 1.5, 3, and 5 kGy irradiated samples, respectively. Considering the non‐significance of pH decrease in minced meat samples, it can be concluded that bacterial activity did not occur. In general, the downward trend of pH during the storage period in meat may be due to proteolysis and decomposition of protein (Otoo, Ocloo, and Appiah 2022), microbial activity, an enhancement of free fatty acids and rancidity reactions that ultimately increase acidity and decrease pH value (Sadakuzzaman et al. 2021). According to the obtained results, it can be said that freezing storage (−18°C) in combination with electron‐beam irradiation largely prevents the occurrence of adverse reactions in minced turkey meat and helps to maintain its quality.

### 3.3 Water Activity

**FIGURE 2:**
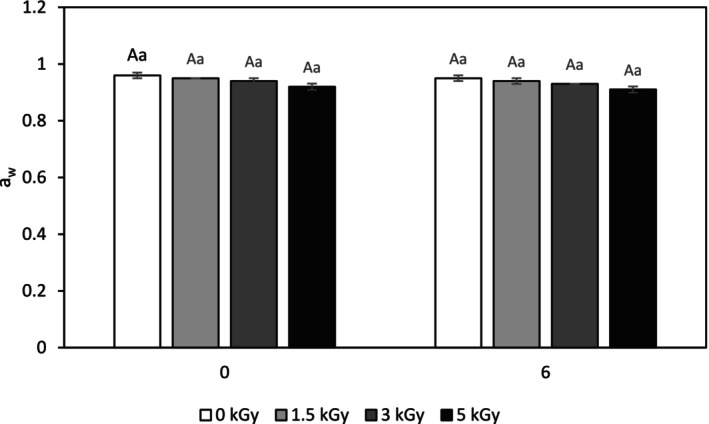
a_w_ of control and electron‐beam irradiated minced turkey meat samples after 6 months storage at −18°C. Different capital letters indicate significant differences among treatments and different small letters indicate significant differences in storage time (*p* < 0.05).

One of the important parameters affecting the shelf life of meat is its water activity (a_w_). a_w_ actually indicates the presence of free water, which accelerates enzymatic processes, especially lipid oxidation, and increases the activity of microorganisms. The determined findings showed that freezing and radiation storage reduces the a_w_ of the samples, although this reduction was not significant (Figure 2). These results allowed supporting the improved microbial characteristics of minced turkey meat samples, which were previously examined in Section 3.1, because the reduction of a_w_ in meat helps to reduce the speed of microbial spoilage processes. A similar finding was obtained by Hassanzadeh et al. (2011) for the effect of gamma irradiation on a_w_ of coated chicken breast meat with chitosan. In addition, Timakova et al. (2018) in their study on the effect of different doses of ionizing radiation on the characteristics refrigerated semi‐finished pork products found that as the dose of radiation increased, a_w_ and microorganism growth decreased.

### 3.4 Peroxide Value

Table 3 details the effect of different irradiation doses and freezing storage (−18°C) on PV (meq peroxide/kg extracted oil from meat) of minced turkey meat. In general moisture content, oxygen exposure, metal ions, high temperature, and UV light can be effective and important in the oxidation of food lipids (Wu et al. 2013). A peroxide index < 5 meq/kg indicates fresh fat, and values of 5–10 meq/kGy indicate the beginning of fat oxidation (Javanmard et al. 2006). When irradiation was applied, the PV of minced meat samples was enhanced with increasing irradiation doses, and the maximum value was obtained for the 5 kGy treated sample. As can be seen, this trend was observed in frozen samples and the irradiation caused a notable enhancement in the PV, and its highest value was achieved in samples that irradiated at 5 kGy after 6 months of storage in −18°C (1.11 meq/kg). However, this obtained PV is much lower than the recommended value (5 meq/kg). It was demonstrated that a radiation dose of < 10 kGy had no remarkable impact on the values of peroxide and iodine of the meats' lipids (Hampson et al. 1996).

**TABLE 3:** Peroxide value (meq/kg) and TBARS (mg/kg) of control and electron‐beam irradiated minced turkey meat samples after storage at −18°C.

Type of analysis	Irradiation doses (kGy)	Storage time (months)	
		0	6
Peroxide value (meq/kg)	0	0.22 ± 0.08^Db^	0.30 ± 0.08^Ca^
	1.5	0.40 ± 0.09^Cb^	0.71 ± 0.06^Ba^
	3	0.61 ± 0.11^Bb^	0.84 ± 0.10^Ba^
	5	0.92 ± 0.09^Ab^	1.11 ± 0.12^Aa^
TBARS (mg/kg)	0	0.08 ± 0.01^Cb^	0.31 ± 0.06^Ca^
	1.5	0.23 ± 0.06^Bb^	0.43 ± 0.05^Ca^
	3	0.48 ± 0.05^Ab^	0.70 ± 0.11^Ba^
	5	0.53 ± 0.05^Ab^	0.85 ± 0.04^Aa^

*Note:* Different capital letters in each column indicate significant differences among treatments, and different small letters in each row indicate significant differences in storage time (*p* < 0.05).

Lipid radicals are created and spread during the intricate process of lipid peroxidation, which also generates various breakdown products. Peroxides are unstable primary oxidation products that, when broken down, produce molecules (secondary products) with quite distinct chemical properties, particularly aldehydes. As hydroperoxides are created during the early stages of lipid peroxidation, PV readings eventually tend to diminish (Sales et al. 2020). Irradiation can cause the formation of hydroxyl radicals in water–oil emulsion systems including meat and meat‐based products, and these radicals can accelerate the reactions of fat oxidation chains. In addition, irradiation initially breaks down the heme pigment in the meat, and as a result, iron released from this pigment or free radicals can catalyze the fat oxidation reaction (Ahn et al. 2004). Ouattara et al. (2002) revealed that oxidation of lipids in beef samples enhances as a result of gamma ray irradiation. Also, Sedeh et al. (2007) found that the PV increased in irradiated beef meat, however, its effect was not significant. Arshad et al. (2019) and Annamalai et al. (2020) earned the same results regarding the irradiation of chicken meat and *Litopenaeus vannamei* , respectively.

### 3.5 TBARS Value

One of the most widely used parameters for assessing lipid oxidation in meat and meat products is the TBARS value (Sojic et al. 2017). It has been proven that the value of TBARS more than 0.5 mg/kg demonstrates partial oxidation (rancidity taste), and when it exceeds 1.0 mg/kg, the meat is severely spoiled and oxidized and is no longer edible (Reitznerová et al. 2017). Table 3 illustrates the TBARS values, as affected by different irradiation dosages as well as storage times. The TBARS content of the nonirradiated sample at time of 0 was 0.08 (mg/kg), which indicates that the used minced turkey meat was fresh and had appropriate quality. As expected, irradiation treatment resulted in higher lipid oxidation, that is, higher TBARS values. It was observed that the TBARS values enhanced notably with increasing radiation dose (*p* < 0.05). Like irradiation, storage in freezing conditions also had a positive impact on increasing the TBARS contents and the highest amount of TBARS values was assessed in 5 kGy irradiated samples after 6 months of freezing storage (−18°C). However, TBARS values of all of the minced meat samples remained below the acceptable limit (1.0 mg/kg) after the storage period. The sensitivity of meat to lipid oxidation and subsequently increase in TBARS values depends on various factors such as storage time, packaging methods, irradiation dose, animal species, and the addition of antioxidant agents. Although free radicals accelerate lipid oxidation in meat, the amount of lipid and fatty acid composition is also very important in meat lipid oxidation during storage time (Kim, Nam, and Ahn 2002).

Many researchers reported that irradiation of meat and meat‐based products intensifies lipid oxidation in them (Asghar et al. 2023; Khalid et al. 2021). Ionizing radiation generates hydroxyl radicals in aqueous systems, and because meat has a high water content, irradiation accelerates oxidative processes in meat. Additionally, exposure to oxygen during storage after irradiation further accelerates lipid oxidation. The combination of oxygen with free radicals formed during radiation creates hydroperoxides. As hydroperoxides decompose, they produce various aldehyde compounds. With higher radiation doses and longer storage times, the TBARS levels in meat samples increase (Derakhshan et al. 2018).

Yan et al. (2024) revealed that irradiation of gamma ray significantly enhances TBARS in fresh pork, which is consistent with our results. In the research of Yu et al. (2022) Atlantic cod was irradiated with doses of 0, 2, 4, 7, and 10 kGy and immediately kept at −20°C for 7 days. They found that TBARS value was greatly enhanced by e‐beam irradiation. Feng et al. (2017) reported that the treatment of ostrich breast meat with electron‐beam irradiation with a dose of 4.5 kGy led to a significant increase in its TBARS content from the initial value of 0.026 to 0.055 (mg/kg). Wahyono et al. (2024) found that the TBARS value increased with increasing electron‐beam irradiation dose and storage time (14 days, 4°C).

### 3.6 Sensory Properties

**FIGURE 3:**
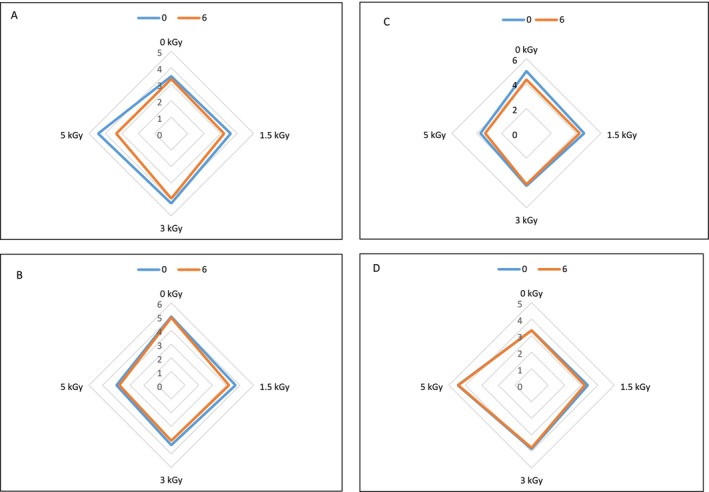
Odor (A), color (B), texture (C), and overall acceptability (D) of control and electron‐beam irradiated minced turkey meat samples during 6 months storage at −18°C.

Sensory properties of minced turkey meat samples at 0 and 6 months after freezing storage (−18°C) were investigated from the aspects of color, odor, texture, and overall acceptability (Figure 3) and minced meat samples that received a sensory score lower than 4 were considered unacceptable for consumption (Majdinasab et al. 2020). The results declared that irradiation dose and storage duration affected sensory characteristics. The highest and the lowest sensory properties of minced meat samples were obtained for control and 5 kGy treated minced meat samples, respectively. Besides, the score of the sensory characteristics, including odor, color, texture, and overall acceptability, decreased over time in all samples. However, 1.5 and 3 kGy treated minced meat samples received an acceptable score even after 6 months of freezing storage (−18°C).

Fallah, Tajik, and Farshid (2010) and Al‐Bachir and Zeinou (2009) also uncovered that the irradiation of camel meat up to 3 kGy has no remarkable impact on the sensory characteristics of samples. Kanatt, Chander, and Sharma (2006) explored the sensory properties of irradiated lamb at 0–5 kGy and their findings are the same as the results of our research. Gomes and da Silva (2006) and Javanmard et al. (2006) observed that the odor and color of chicken meat were not significantly influenced by irradiation treatment. In a research carried out on shrimp, the results showed that irradiation of shrimp meat with a dose of 6 kGy did not have a negative impact on the sensory parameters of the meat sample, and all its parameters such as smell and taste were acceptable (Wang et al. 2010). Kanatt, Chander, and Sharma (2005), also reported that irradiation treatment of sheep, pork, and chicken meat with doses of 1–5 kGy increased the safety quality and duration of meat storage in cold conditions for more than 2 weeks compared to the control group, without significant effect on its characteristics.

It has been reported that the use of the irradiation process in doses < 10 kGy improves the toxicological and microbial safety of foods, without having a negative effect on their sensory quality (Hashim, Yusop, and Rahman 2024). However, the results obtained by Dini et al. (2020) showed that irradiating beef loins with gamma rays at 2.5 kGy significantly reduced the score of sensory characteristics such as odor and overall acceptability. It seems that changes in sensory characteristics of irradiated samples are influenced by parameters such as the type of meat, irradiation source, ray dose, exposure to oxygen (during and/or after the radiation process), temperature during radiation, presence of antioxidants, and type of packaging (Brewer 2009).

## 4 Conclusion

In conclusion, the combination of freezing storage (−18°C) and irradiation can be considered an effective way to remove pathogens from minced turkey meat and improve its microbial quality and safety. However, the mesophilic and psychrotrophic bacteria enumeration reduced remarkably after irradiation process and freezing storage, the pH and a_w_ of samples were not affected significantly by these treatments. Although, the irradiation process and storage at −18°C increased TBARS and PV values of minced meat samples, 1.5 and 3 kGy treated minced meat samples received an acceptable sensory score on the last day of the storage period. By applying combination (Hurdle) technology, the minced turkey meat shelf life can be improved by up to 6 months without having an adverse influence on the chemical and sensory characteristics. Considering that high doses of radiation lead to an increase in fat oxidation of minced meat samples, a dose of 3 kg is suggested for minced turkey meat irradiation to improve its storage safety and shelf life.

## Author Contributions

**Mahdieh Salari:** project administration (lead), supervision (lead), writing – original draft (lead), writing – review and editing (lead). **Moein Khatami:** data curation (equal), investigation (lead), methodology (lead).

## Ethics Statement

The authors have nothing to report.

## Consent

Written informed consent was obtained from all participants in the study.

## Conflicts of Interest

The authors declare no conflicts of interest.

## Data Availability

All the data used is shown in this article.
